# Supplementary material for: Megathrust reflectivity reveals the updip limit of the 2014 Iquique earthquake rupture
Source: Nat Commun. 2022 Jul 8;13:3969. doi: 10.1038/s41467-022-31448-4 (PMC9270347; doi:10.1038/s41467-022-31448-4)
Supplement: Supplementary file 1 — Supplementary Information [file 41467_2022_31448_MOESM1_ESM.pdf]

Supporting information

"Megathrust reflectivity reveals the updip limit of the 2014 Iquique earthquake rupture"

Bo Ma[1*], Jacob Geersen[1,2], Dietrich Lange[1], Dirk Klaeschen[1], Ingo Grevemeyer[1,2], Eduardo Contreras-Reyes[4], Florian Petersen[1], Michael Riedel[1], Yueyang Xia[1*], Anne M. Tréhu[3], and Heidrun Kopp[1,2]

[1] GEOMAR Helmholtz Centre for Ocean Research Kiel, Germany

[2] Institute of Geosciences, Kiel University, Kiel, Germany

[3] Oregon State University, College of Earth, Ocean, and Atmospheric Sciences, Corvallis, USA

[4] Departamento de Geofísica, Facultad de Ciencias Físicas y Matemáticas, Universidad de Chile, Santiago, Chile

* Corresponding author: B. Ma (bma@geomar.de), Y. Xia (yxia@geomar.de)

1. Seismic Processing

Seismic data processing was conducted with the Schlumberger OMEGA2 software. Here we present the processing sequences and the corresponding results in terms of seismic line MC04. The same processing strategy is used for all profiles to eliminate the possibility that differences in reflectivity are due to differences in processing. An overview of the processing scheme is shown below (Suppl. Fig. 1). The main seismic processing included four steps:

A. Step1: Processing Geometry Implementation

In this step, the seismic data are exposed to a series of pre-processing modules of the Schlumberger OMEGA2 software, including sail extract module, geometry crooked module, geometry update module and grid define module, aiming to implement the processing geometry. In particular, the sail extract module is used to merge the geometry survey file information with the seismic traces. The geometry crooked module analyses the location of detectors, sources, and midpoints and projects them onto a smooth 2-D crooked common-mid-point (CMP) profile. Subsequently, the geometry update module and the grid define module were used to update the seismic trace header literals to the new processing grid. These pre-processing steps prepare the seismic data set for further analysis. Seismic shooting was conducted at an interval of 125 m to avoid interference from the previous shot on large offset OBS data. Due to the large shot interval, the original CMP gathers are imaged with prominent spatial aliasing. The aliasing has serious effects on the performance of multichannel data analysis processes such as f-k filtering. Because of spatial aliasing, these processes can perceive events with steep dips at high frequencies as different from what they are and, hence, do not treat them properly.

B. Step2: Increase Vertical Resolution and General Noise Cleaning

In this step, four modules were used to increase the vertical resolution and clean the general noise. In particular, the deconvolution module is applied to compress the basic wavelet, thus increasing temporal resolution. The direct wave cleaning module was employed in shallow seafloor areas to enhance near-surface wide-angle reflections. Attenuation of random noise (RNA) was applied by a predictive deconvolution in the fx-domain. The anomalous amplitude attenuation (AAA) removed high amplitude events such as marine swell, rig and ship noise by transforming the seismic data into frequency bands and applying a spatial median filter. These pre-processing steps yield CMP gathers (Suppl. Fig. 2a) which will be interpolated in the next step.

C. Step3: Shot Interpolation

To avoid prominent spatial aliasing between neighboring shots resulting from the large shot interval, shot-ordered seismic data were interpolated three times from 125 m to 15.625 m by f-k trace interpolation. As the result is not a multiple of the receiver interval, an irregular interpolator module was used to interpolate it to 12.5 m, where aliased components have been eliminated (Suppl. Fig. 2b).

D. Step 4: Multiple attenuation

Prior to multiple attenuation, the plate boundary reflection is severely obscured by multiples (Suppl. Fig. 3a). The multiples were predicted by surface related multiple elimination (SRME) using wavefield inversion based on the Kirchhoff integral and subtracted from the raw data after shot interpolations. After the multiple attenuation, we applied the f-k filtering to remove the high frequencies to enhance the image (Suppl. Fig. 3b). The plate boundary reflection is clearly observed in the travel time range where the first multiple occurs and we can now trace the plate boundary interface further landward on the time section of seismic line MC04 (Suppl. Fig. 4). The extensive data volume generated by the shot interpolations led to long calculation times in the pre-stack time domain and the pre-stack depth domain, so we reconstructed the offsets of CMP gathers with an increment of 100 m. After the reconstruction of offset distances, a preliminary v_p model based on an unpublished 3D v_p model from ocean bottom seismometers (OBS) during cruise MGL1610 of the *R/V Marcus G. Langseth* in 2016 was applied for the pre-stack depth migration. Referring to the OBS velocity field, we calculated the velocity gradient starting from the seafloor to yield an initial velocity model (Suppl. Fig. 5) for application to the multichannel seismic profiles. Although this initial velocity model is not sufficiently confining the shallow depth (< 8 km below seafloor) and may produce an inaccuracy of 1-2 km in the depth of the plate boundary compared to an accurate velocity model, it does not affect the spatial distribution of the reflectivity. In the meanwhile, this initial velocity does not enable to carry out amplitude analysis at shallow depth. However, our main focus is on the reflectivity pattern and the lateral coherence and continuity of the plate boundary

reflectivity. The pre-stack depth migrated seismic images are not significantly affected by seafloor multiples anymore and resolve sub-seafloor structures in the upper plate and subducting lower plate at high resolution (Suppl. Fig. 6).

E. Step 5: Amplitude calibration

To quantify the amplitudes of the seismic sections, the reflection coefficient was estimated based on the ratio of the seafloor reflection to the seafloor multiple reflection. Due to interference of several reflector elements resulting in inverse and mix phased signals especially in the crustal overburden the envelope was calculated representing the absolute reflection strength (Suppl. Fig. 7). The plate boundary shows a unique reflection strength of 0.005 to 0.0075 in a depth range between 15 to 35 km, whereas the internal crustal reflector elements show a higher variability ranging from 0.0075 to 0.025 at a depth range of 5 to 15 km.

2. Thermal Model Setup

It has long been recognized that the rupture zone of subduction zone megathrust earthquakes is at least partially controlled by the thermal state of the fault zone[1,2]. Analytical models reveal that the geometry of the subduction zone, the thermal state of the incoming subducting plate and the shear or frictional heating along the megathrust are critical parameters controlling megathrust temperatures[3,4], which in turn, define the seaward and landward limit of large subduction earthquakes rupture zones. The seaward or updip limit is generally associated with temperatures of 100°-150°C, marking the smectite to chlorite transition[2] or a suite of diagenetic reactions and release of water from underthrusted sediments[5] and/or basement[6]. The landward or downdip limit is assumed to be associated with a critical temperature of 350°-400°C, which marks the transition from stick-slip to stable sliding at the onset of quartz and feldspar plasticity of continental crustal rocks[7].

The geometry of the subduction zone is readily known from geophysical data or the hypocentral depth of large megathrust earthquakes and the basal heat flow is defined by the age

of the incoming oceanic plate. Most thermal parameters of subduction zones show little variation along the Pacific Ring of Fire and are well established, especially for Chile[8,9]. Based on these data, most subduction zones have been studied using two-dimensional thermal models. Here, we use two different approaches. First, we calculate the thermal state along the plate interface or megathrust fault using analytical solutions based on the formalism of ref.[4] and second, we consider a 2-dimensional thermal model of ref.[10] for northern Chile, incorporating corner flow in the mantle wedge.

Analytical expressions, which relate surface heat flux to temperature, geometrical constraints, and shear stress, provide an efficient approach to study the thermal state of the megathrust fault and are discussed in detail by refs.[3,4,11]. We follow the approach of ref.[4] and first calculate the temperature T_f as a function of the depth z on the interplate fault zone as:

$$(1)\ T(z) = K_m\, T_0\, z / S K_s [\pi \kappa (t_0 + t_s)]^{1/2}$$

where $S(z) = 1 + bK_m[(V_n\, z\, \sin\delta)/\kappa]^{1/2} / K_s$. K_m (3.3 $Wm^{-1}K^{-1}$) and K_s (2.55 $Wm^{-1}K^{-1}$) are the mantle and forearc thermal conductivity, respectively. T_0 is the asthenospheric mantle temperature (1300°C) and κ is the thermal diffusivity ($10^{-6} m^2 s^{-1}$). t_0 is the average age of the subducting plate (50 Myr[12]), whereas t_s is the time it takes the lithosphere to subduct to a depth z. V_n is the convergence rate normal to the subduction (~70 km/Myr), δ is the dip angle of subduction, and $b(\pi^{-1/2})$ is a factor that depends on the specific geometry[11].

The dip angle of subduction was taken directly from the seismic reflection images (Fig. 2), and the time t_s is computed by dividing the integrated downdip length of the fault surface by V_n[13]. We neglect the effect of the horizontal heat flow. To calculate the radiogenic heat production T_r in the forearc crust we used the following expression[13]:

$$(2)\ T_r(z) = A_r\, z^2 / (2K_s\, S(z))$$

where A_r is the radiogenic heat production rate (10^{-6} Wm^{-3})[14]. Radiogenic heat production adds 0-45°C to the fault temperature from the trench axis up to the downdip limit.

Further, we include in our model frictional shear heating $T_{sh}(z)$ on the thermal field by using:

$$(3)\ T_{sh}(z) = \tau(z) V_t\, z/(K_s\, S(z))$$

where $\tau(z)$ is the shear stress on the fault and V_t is the total slip rate[4,11]. $\tau(z)$ on a gently dipping fault at shallow depth is approximately

$$(4)\ \tau(z)=\mu(\sigma_n(z)-p(z))$$

where μ is the friction coefficient, σ_n is the normal stress applied on the fault plane (approximately the overburden pressure), and p is the pore fluid pressure. Following ref.[13], we use $\mu = 0.85$, $\sigma_n(z)=\rho g z$ and $p(z)=0.95\sigma_n(z)$ with acceleration of gravity g=9.8 ms^{-2} and the average crustal density ρ=2500 kgm^{-3}.

The final predicted temperature on the fault plate boundary $T_f(z)$ is the sum of Eqs. (1)-(3) (i.e., $T_f(z)= T(z)+ T_r(z)+ T_{sh}(z)$). Fig. 2 shows the estimated temperature values for $T_f(z)$ along our seismic reflection lines.

We compare our model to the numerical model of ref.[10]. The geometry of this model is based on a suite of geophysical data[15] and thermal parameters were rated against a number of observed features, including the maximum depth of subduction thrust earthquakes and observed heat flow. Interestingly, the maximum depth of seismic faulting of megathrust earthquakes in northern Chile occurs at 40-50 km[16,17], suggesting that temperatures of 350°-400°C are reached at ~40-50 km, too. To mimic this feature, ref.[10] had to introduce a considerable amount of shear heating, in the order of τ=33 MPa to τ=67 MPa, with the upper limit providing a better fit to the data. The predictions from the τ=67 MPa model mimic the prediction of our preferred analytic solution down to a depth of approx. 30 km. At greater depth, the models differ with the ref.[10] model showing somewhat lower temperatures. The observed differences may stem from the effects of the asthenospheric corner flow incorporated into the numerical model and a

change in dip angle, which is not considered in the analytic model. We also compare our model with other thermal models from the Northern Chilean margin. A comparison of all the models for the temperature along the plate interface is shown in Suppl. Fig. 9, including the thermal model with shear stress τ=67 MPa[10] as the black dashed line, the thermal model with frictional heating we used in the main text as the red dashed line, the thermal model with shear stress τ=33 MPa[10] as the orange dashed line, the thermal model of ref.[18] as the magenta dashed line, and a thermal model we built without the frictional heating as the green dashed line. Please note that for the study of the up-limit limit at shallow depth (~15 km) and its correlation to the reflectivity pattern of the seismic data, the model we established with frictional heating and the model with shear stress τ=67 MPa of ref.[10] show consistent features.

As previously mentioned in the main text, the established new thermal model uses a friction coefficient μ=0.85 and pore fluid pressure λ=0.95[13]. Since these values vary in each tectonic setting, we applied different μ_b in the new analytical thermal model in Suppl. Fig. 10. The effective coefficient of basal friction μ_b depends on both the friction coefficient μ and pore fluid pressure λ along the fault zone: $\mu_b = \mu(1-\lambda)$[19]. Based on this formula, the μ_b of the model in our main text is 0.0425, which is shown as the red dashed line. We applied a range of μ_b from 0.03-0.13, consistent with the global thermal measurement[20]. In this range, the predictions depth of the analytical model (< 20 km) is close to the downdip limit of the megathrust reflectivity (~15 km) observed from MCS images at the upper threshold of the clay dehydration temperature of 150°C (Suppl. Fig. 10). Moreover, the thermal model and reflectivity show a better spatial matching with a higher effective coefficient of basal friction μ_b value.

An interesting feature is that the models for northern Chile show larger frictional heating compared to those observed in south-central Chile[8,9,14]. However, already ref.[9] suggested that frictional heating at the plate boundary increases northward, perhaps mimicking the increasing age of the subducting plate. Furthermore, patterns are consistent with heat flow anomalies over

the marine forearc. Heat flow anomalies over the marine forearc are in the order of 50-60, 40-50, and 24-31 mW/m^2 at 39°S, 36°S and 33°S, respectively[14], decreasing northward and hence reflecting increasing crustal age of the subducting plate and supporting a decrease of basal heat flow. At 21°S, however, the age of the subducting plate has increased by roughly 20 Myr with respect to 33°S, but the forearc heat flow is in the order of 30-40 mW/m^2[1] and thus higher than near 33°S, supporting higher values frictional heating than found further south. It might be reasonable to hypothesize that sediment starved subduction erosion supports a higher degree of friction than the accretionary margin of south-central Chile, but this interpretation is beyond the scope of our work.

Both our analytical model and the numeric 2D model of ref.[10] show higher temperatures along the subduction megathrust fault with respect to other models for northern Chile. For example, ref.[18] (Suppl. Fig.8, magenta dashed line) did not consider any frictional heating with the argument that in south-central Chile shear heating was low and therefore they obtained lower temperatures.

3. Supplementary Discussion

Reflection energy absorption, seismic processing parameters, differences of gun energy during seismic acquisition and shooting direction may all potentially cause a variation of reflectivity along the plate boundary. To allow a spatial comparison of the reflectivity and avoid issues caused by seismic processing, the same processing strategy is used for all profiles. In addition, we discuss the following issues:

A. Is the plate interface on seismic line MC04 visible to greater depth because of stronger gun energy during data acquisition?

The plate interface is traced to a depth of approximately 35 km on the northern dip line MC04 but disappears at shallower depth on the dip lines that run through the rupture area of

the 2014 Iquique earthquake. The acquisition geometry and gun array remained unchanged during the survey, which covered seismic lines parallel to the trench as well as profiles in the dip direction. Strike line MC30, which crosses the middle continental slope and is located furthest from the trench axis, documents that the plate interface along the northern part of the line can be traced to greater depth than along the southern part. This observation is augmented by all seismic dip lines and seismic strike lines of our survey.

B. Is the difference in the plate interface reflection strength caused by shooting direction?

The shooting direction did not cause the observed differences of the plate interface reflection as seismic dip lines MC04, MC 06, and MC25 were shot from west to east, while seismic dip lines MC17 and MC23[22] were shot from east to west. There are at least two seismic lines in the same direction, documenting that the shooting direction does not exert a major influence.

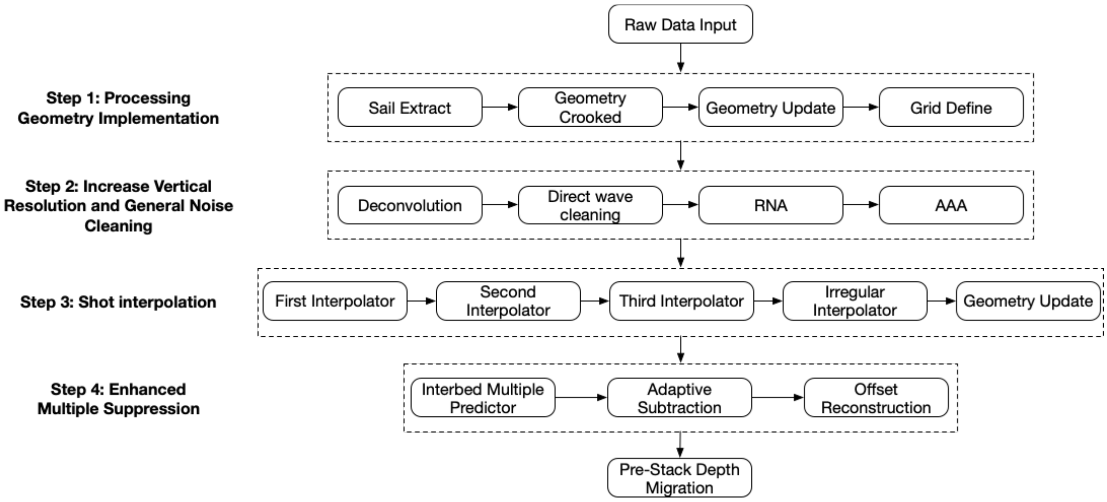

Suppl. Fig. 1: Overview of the processing sequence.

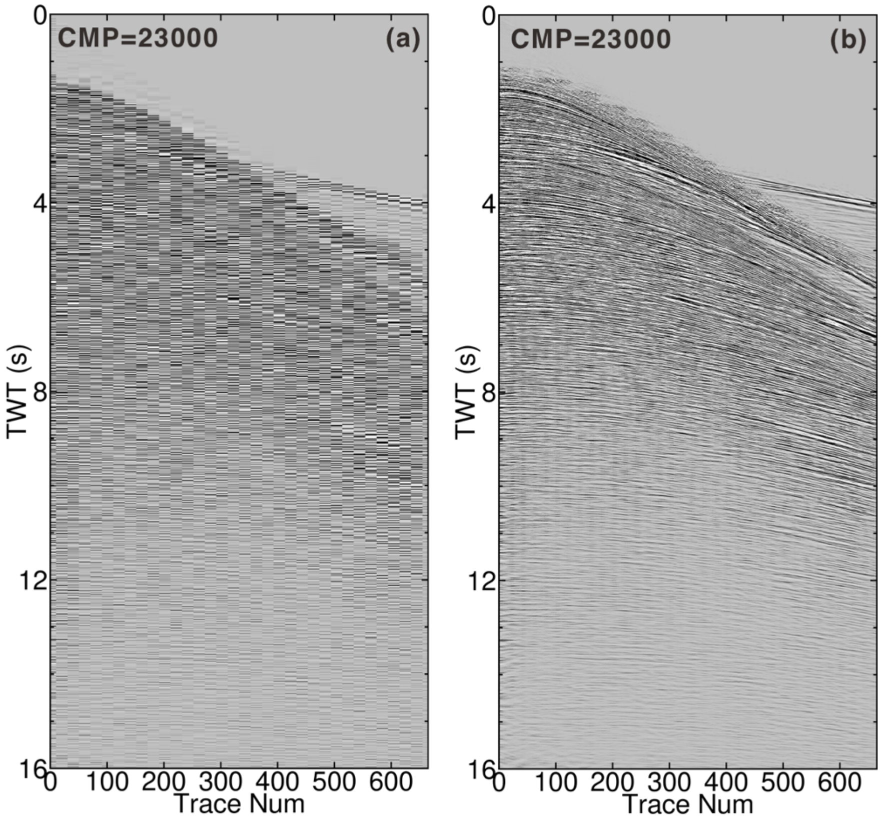

Suppl. Fig. 2: The CMP gathers before and after interpolation of seismic line MC04. Before the interpolation processing, the trace spacing is 250 m, which is shown in (a). After the interpolation processing the trace spacing is 25 m in (b).

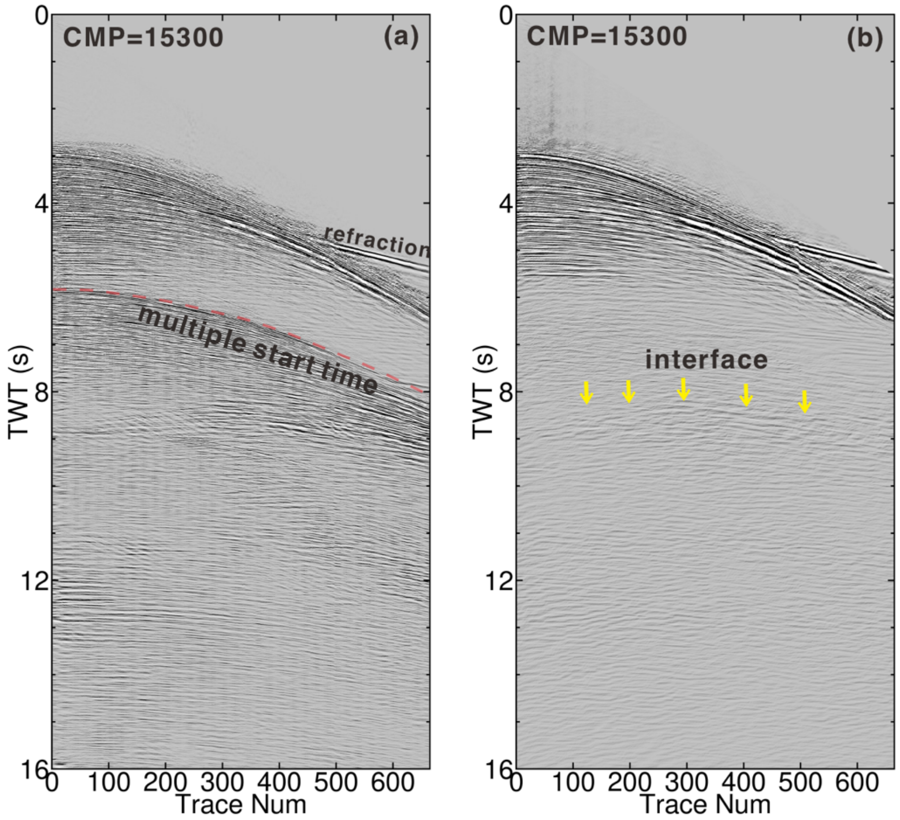

Suppl. Fig. 3: The CMP gathers before and after the de-multiple step of MC04. Before the demultiple processing, seismic signals are obscured by several multiple orders below the seafloor multiple start time indicated by the transparent red dashed line in (a). After the demultiple processing and high frequencies elimination, the multiples are mostly eliminated and we can see the interface traced by yellow arrows around 8-9 s TWT in (b).

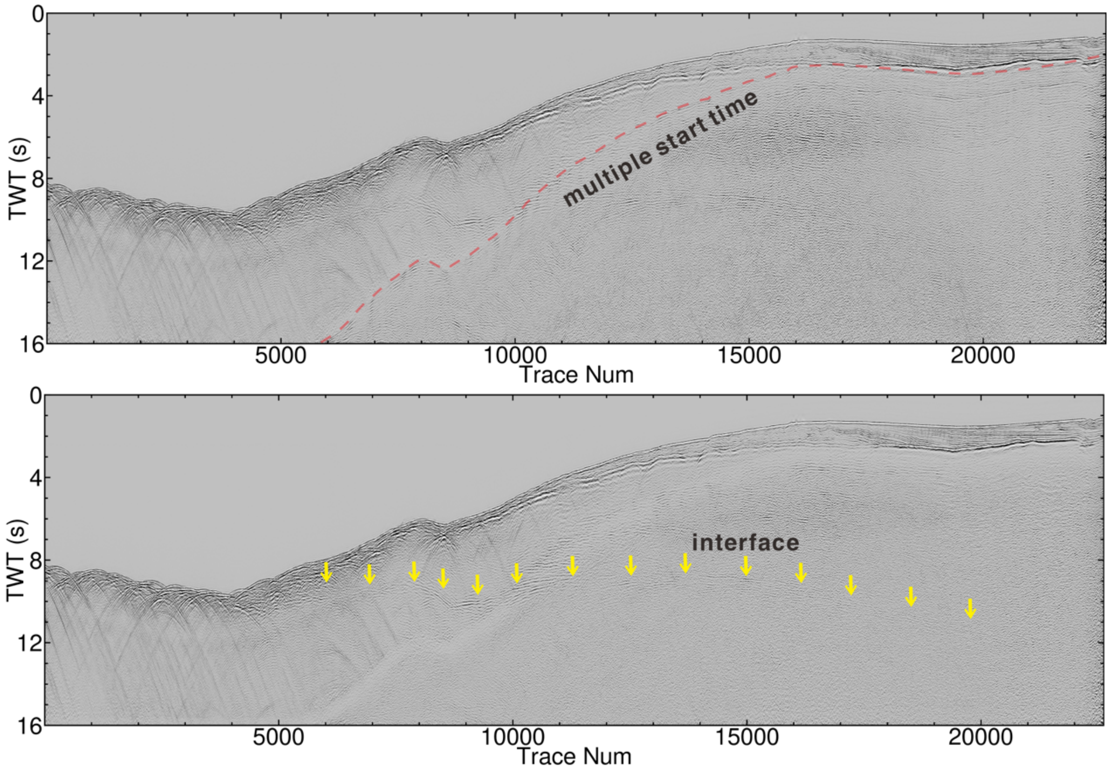

Suppl. Fig. 4: Stack section in the time domain of seismic line MC04 before and after the demultiple processing. Before the demultiple processing, the interface reflection is obscured by the multiples below the seafloor multiple start time indicated by the transparent red dashed line in (a). After the demultiple processing, the interface reflection can be traced around trace number 20000, as indicated by the yellow arrows in (b). Sections are shown after application of a depth customized gain of amplitudes.

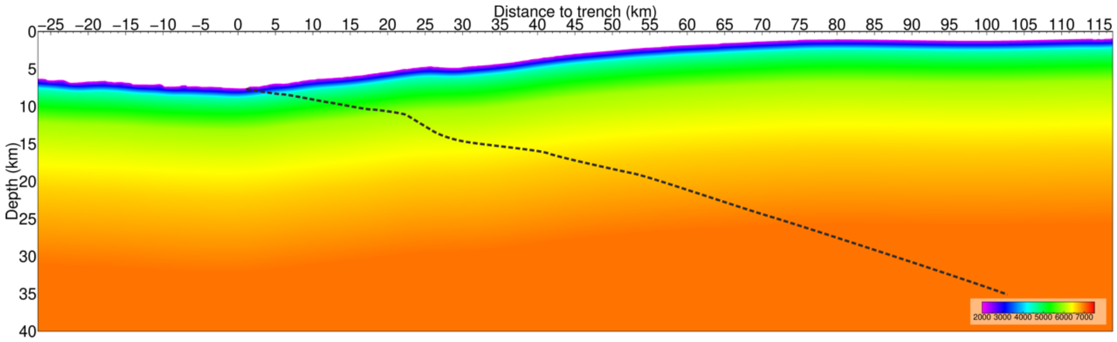

Suppl. Fig. 5: An initial velocity model based on an unpublished 3D OBS velocity model (K. Davenport, personal communication) was applied for the pre-stack depth migration. The black dashed line indicates the plate boundary of profile MC04.

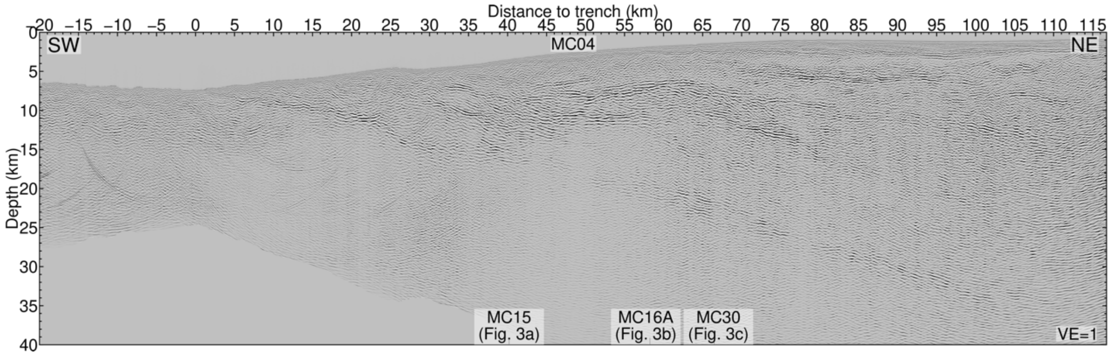

Suppl. Fig. 6: The pre-stack depth migrated seismic image of seismic line MC04.

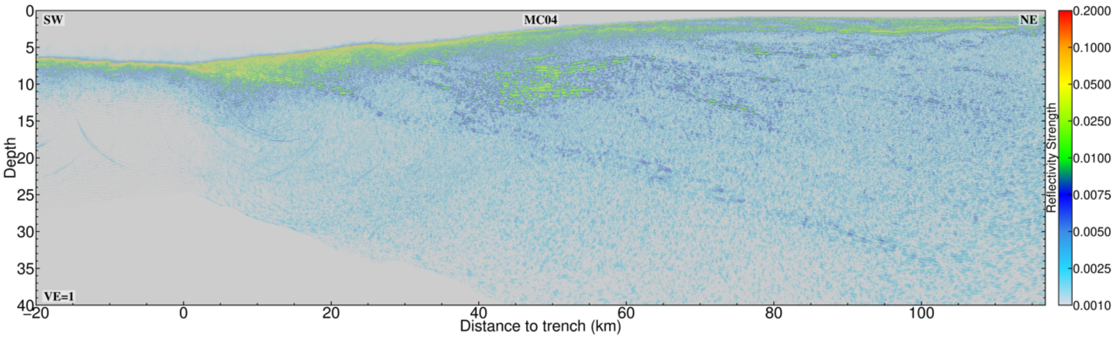

Suppl. Fig. 7: Estimated reflection strength of pre-stack depth migrated seismic image of seismic line MC04. The plate boundary shows a unique reflection strength than the internal crustal reflector elements. Vertical exaggeration is 1.

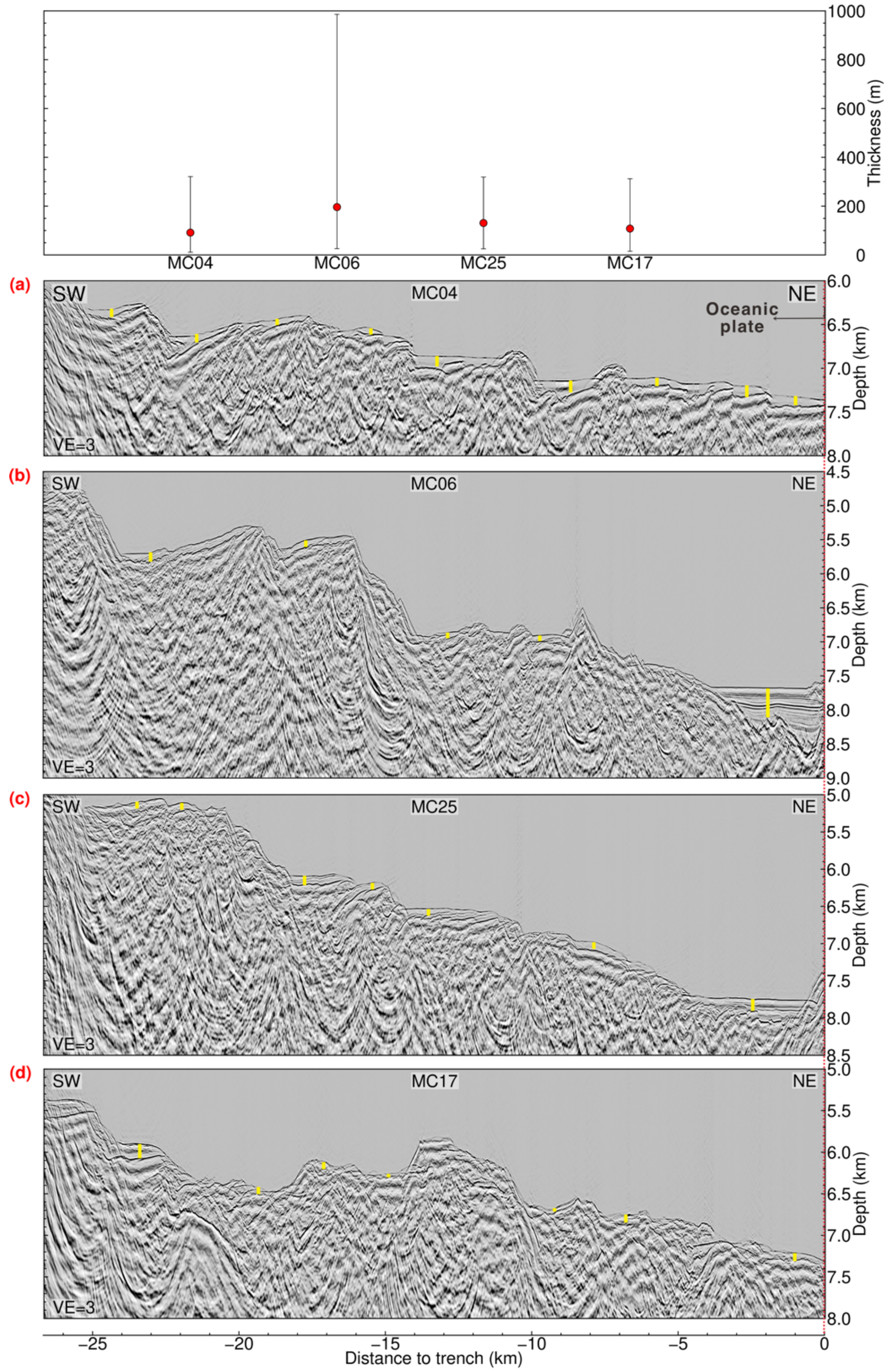

Suppl. Fig. 8: Oceanic crust of pre-stack depth migrated section along seismic dip-lines. The error bar on top indicates the sediment upon the oceanic crust, in which the red dots show the average thickness of sediment on oceanic crust. The upper limit of the error bar represents the maximum thickness of sediments, while the lower limit indicates the minimum thickness of sediment. The maximum thickness anomaly along MC06 is due to more sediment in the trench than along the other seismic lines. In (a)-(d), yellow solid lines indicate the thickness of

sediment on oceanic crust. Due to the bending of the oceanic crust, the sediments are accumulated in half-graben structures. (a): seismic line MC04; (b): seismic line 06; (c): seismic line 25; (d): seismic line 17. Vertical exaggeration is 3.

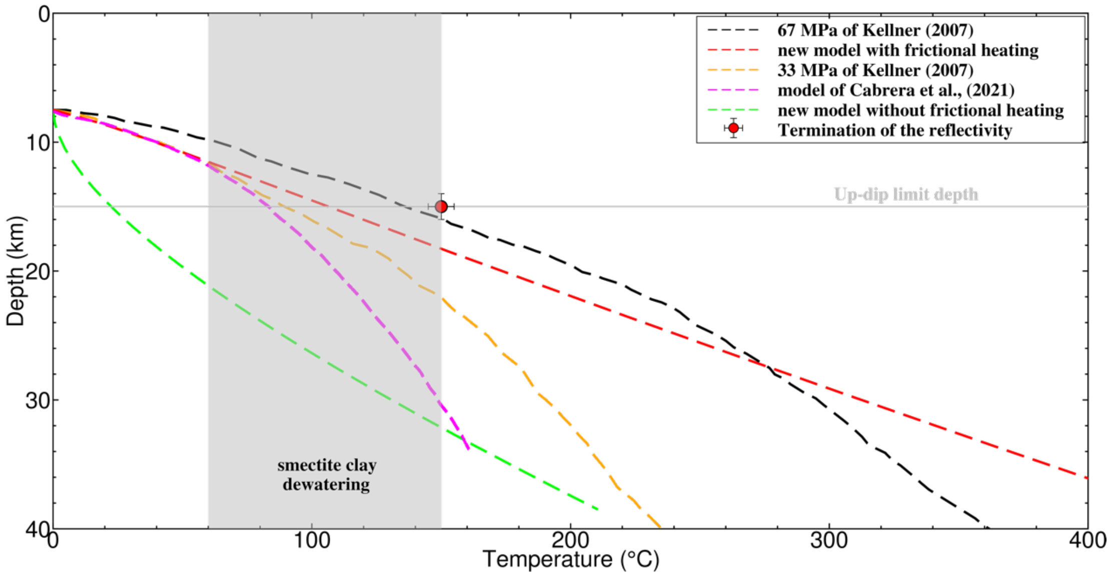

Suppl. Fig. 9: Comparison of different thermal models. Black dashed line: thermal model with shear stress τ=67 MPa[10]; red dashed line: thermal model with frictional heating discussed in main text; orange dashed line: thermal model with shear stress τ=33 MPa[10]; magenta dashed line: thermal model of ref.[18]; green dashed line: alternative thermal model computed without frictional heating. The approximate depth range of smectite clay dehydration is based on refs[5,23-26].

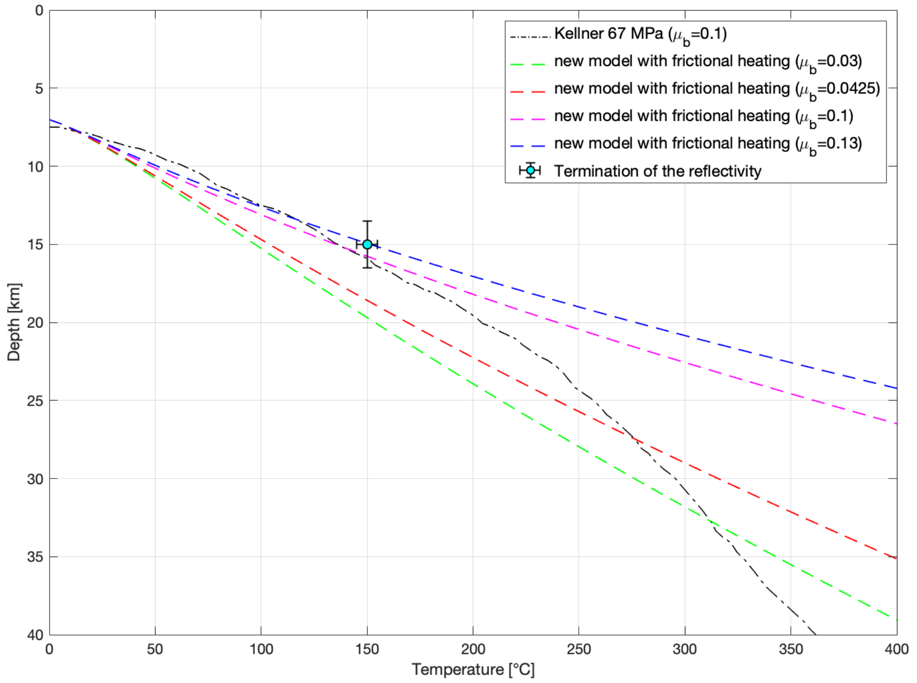

Suppl. Fig. 10: New thermal model with varying effective coefficient of basal friction μ_b values. The range of μ_b is from 0.03-0.13, consistent with the global thermal measurement[20]. The estimated average basal friction μ_b=0.1 of Kellner 67 MPa is shown as a black dashed line. Using the same basal friction value, our new analytical model is shown as a magenta dashed line. In the main text, we used a friction coefficient of μ=0.85 and pore fluid pressure λ=0.95[13]. The effective coefficient of basal friction μ_b depends on both the friction coefficient μ and pore fluid pressure λ along the fault zone: $\mu_b = \mu(1-\lambda)$[19]. Based on this formula, the μ_b of the model in our main text is 0.0425, which is shown as red dashed line. μ_b=0.03 and μ_b=0.13 are shown as green and blue dashed lines, respectively.

References

1. Tichelaar, B. W. & Ruff, L. J. Depth of Seismic Coupling Along Subduction Zones. J. Geophys. Res. **98**, 2017-2037 (1993).
2. Hyndman, R. D. & Wang, K. Thermal Constraints on the Zone of Major Thrust Earthquake Failure - the Cascadia Subduction Zone. J. Geophys. Res. **98**, 2039-2060 (1993).
3. Molnar, P. & England, P. Temperatures, Heat-Flux, and Frictional Stress near Major Thrust Faults. J. Geophys. Res. **95**, 4833-4856 (1990).

4. England, P. On Shear Stresses, Temperatures, and the Maximum Magnitudes of Earthquakes at Convergent Plate Boundaries. J. Geophys. Res. **123**, 7165-7202 (2018).
5. Spinelli, G. A. & Saffer, D. M. Along-strike variations in underthrust sediment dewatering on the Nicoya margin, Costa Rica related to the updip limit of seismicity. Geophys. Res. Lett. **31**, L04613 (2004).
6. Kameda, J. *et al.* A new source of water in seismogenic subduction zones. Geophys. Res. Lett. **38**, L22306 (2011).
7. Scholz, C. H. The Brittle-Plastic Transition and the Depth of Seismic Faulting. Geol. Rundsch. **77**, 319-328 (1988).
8. Völker, D., Grevemeyer, I., Stipp, M., Wang, K. & He, J. Thermal control of the seismogenic zone of southern central Chile. J. Geophys. Res. **116**, B10305 (2011).
9. Rotman, H. M. M. & Spinelli, G. A. Remarkably consistent thermal state of the south central Chile subduction zone from 36 degrees S to 45 degrees S. J. Geophys. Res. **119**, 3503-3516 (2014).
10. Kellner, A. Different styles of deformation of the fore-arc wedge along the Chilean convergent margin: Insights from 3D numerical experiments. *https://doi.org/10.2312/GFZ.b103-07113* (Deutsches GeoForschungsZentrum GFZ, Potsdam, 2007).
11. Molnar, P. & England, P. Temperatures in Zones of Steady-State Underthrusting of Young Oceanic Lithosphere. Earth Planet. Sci. Lett. **131**, 57-70 (1995).
12. Muller, R. D., Sdrolias, M., Gaina, C. & Roest, W. R. Age, spreading rates, and spreading asymmetry of the world's ocean crust. Geochem. Geophys. Geosyst. **9** (2008).
13. McCaffrey, R., Wallace, L. M. & Beavan, J. Slow slip and frictional transition at low temperature at the Hikurangi subduction zone. Nat. Geosci. **1**, 316-320 (2008).
14. Grevemeyer, I., Diaz-Naveas, J. L., Ranero, C. R., Villinger, H. W. & Sci, O. D. P. L. Heat flow over the descending Nazca plate in central Chile, 32 degrees S to 41 degrees S: observations from ODP Leg 202 and the occurrence of natural gas hydrates. Earth Planet. Sci. Lett. **213**, 285-298 (2003).
15. Oncken, O. *et al.* Seismic imaging of a convergent continental margin and plateau in the central Andes (Andean Continental Research Project 1996 (ANCORP' 96)). J. Geophys. Res. **108** (2003).
16. Tichelaar, B. W. & Ruff, L. J. Seismic Coupling Along the Chilean Subduction Zone. J. Geophys. Res. **96**, 11997-12022 (1991).
17. Peyrat, S. *et al.* Kinematic rupture process of the 2007 Tocopilla earthquake and its main aftershocks from teleseismic and strong-motion data. Geophys. J. Int. **182**, 1411-1430 (2010).
18. Cabrera, L. *et al.* Northern Chile intermediate-depth earthquakes controlled by plate hydration. Geophys. J. Int. **226**, 78-90 (2021).
19. Hubbert, M. K. & Rubey, W. W. Role of Fluid Pressure in Mechanics of Overthrust Faulting: 1. Mechanics of Fluid-Filled Porous Solids and Its Application to Overthrust Faulting. Geol. Soc. Am. Bull. **70**, 115-166 (1959).
20. Gao, X. & Wang, K. Strength of stick-slip and creeping subduction megathrusts from heat flow observations. **345**, 1038-1041 (2014).
21. Springer, M. & Forster, A. Heat-flow density across the Central Andean subduction zone. Tectonophysics **291**, 123-139 (1998).
22. Petersen, F. *et al.* Relationship Between Subduction Erosion and the Up-Dip Limit of the 2014 Mw 8.1 Iquique Earthquake. Geophys. Res. Lett. **48**, e2020GL092207 (2021).
23. Saffer, D. M. & Tobin, H. J. Hydrogeology and Mechanics of Subduction Zone Forearcs: Fluid Flow and Pore Pressure. Annu. Rev. Earth Planet. Sci. **39**, 157-186 (2011).
24. Kastner, M., Elderfield, H. & Martin, J. B. Fluids in Convergent Margins - What Do We Know About Their Composition, Origin, Role in Diagenesis and Importance for Oceanic Chemical Fluxes. Philos. Trans. Royal Soc. A. **335**, 243-259 (1991).
25. Bekins, B., Mccaffrey, A. M. & Dreiss, S. J. Influence of Kinetics on the Smectite to Illite Transition in the Barbados Accretionary Prism. J. Geophys. Res. **99**, 18147-18158 (1994).
26. Underwood, M. B. Sediment Inputs to Subduction Zones: Why Lithostratigraphy and Clay Mineralogy Matter. in *The seismogenic zone of subduction thrust faults.* 42-85 (Columbia University Press, 2007).
